# Supplementary material for: Genetic variation and DNA fingerprinting of durian types in Malaysia using simple sequence repeat (SSR) markers
Source: PeerJ. 2018 Mar 2;6:e4266. doi: 10.7717/peerj.4266 (PMC5836569; doi:10.7717/peerj.4266)
Supplement: Data S1 [file peerj-06-4266-s002.pdf]

Supplementary Data – DNA fingerprinting gel images

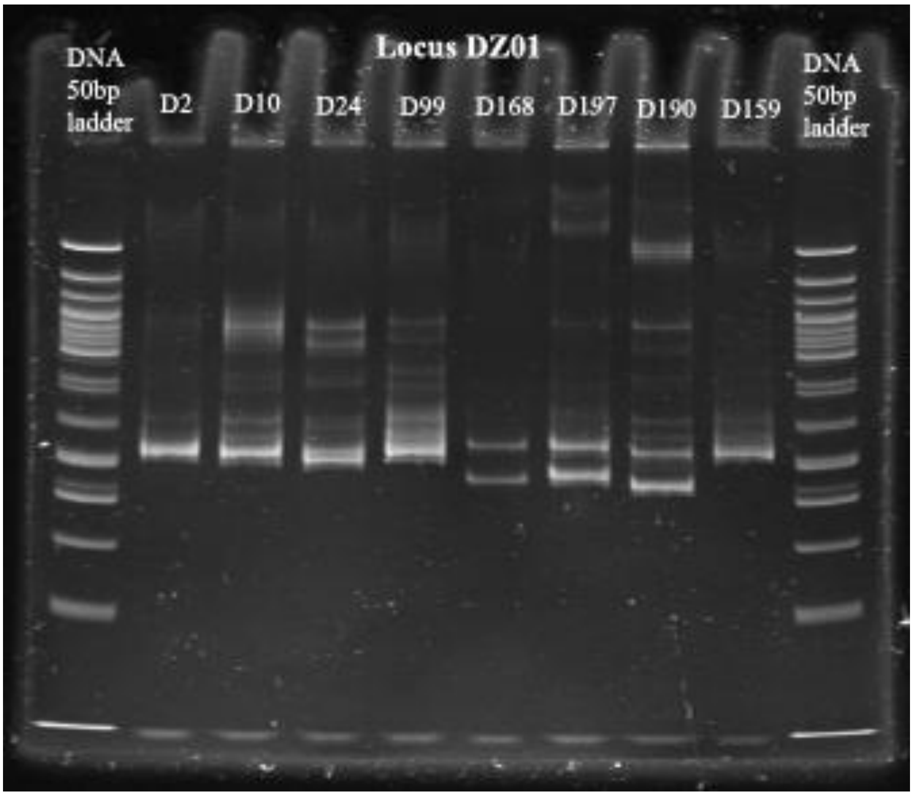

Figure S1 PCR products of eight durian types at locus DZ01 on 8% (w/v) polyacrylamide gel

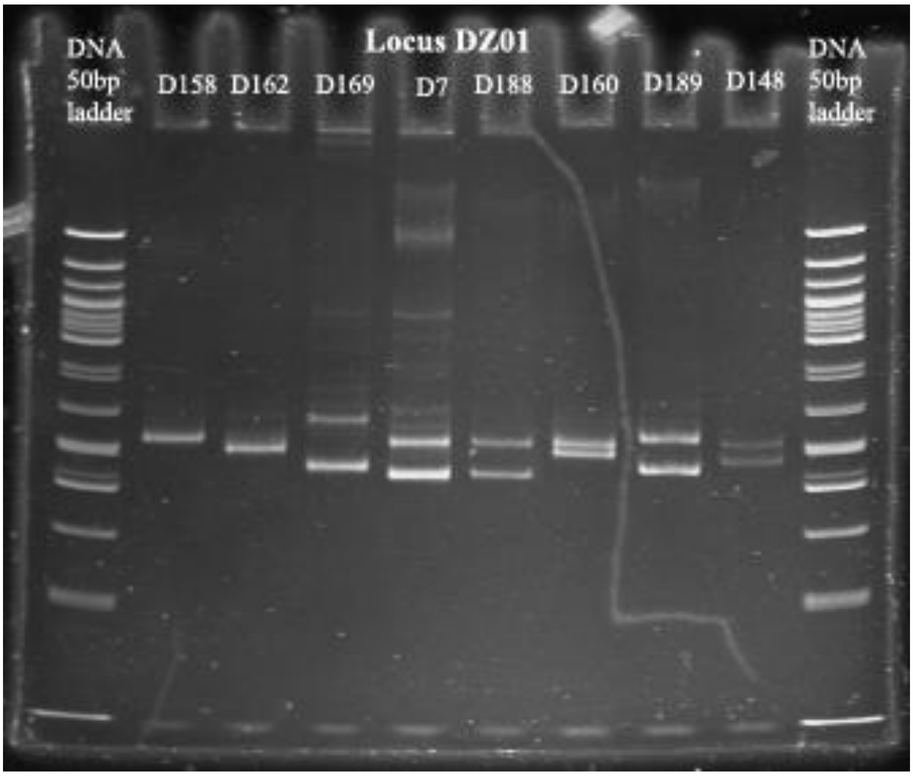

Figure S2 PCR products of eight durian types at locus DZ01 on 8% (w/v) polyacrylamide gel

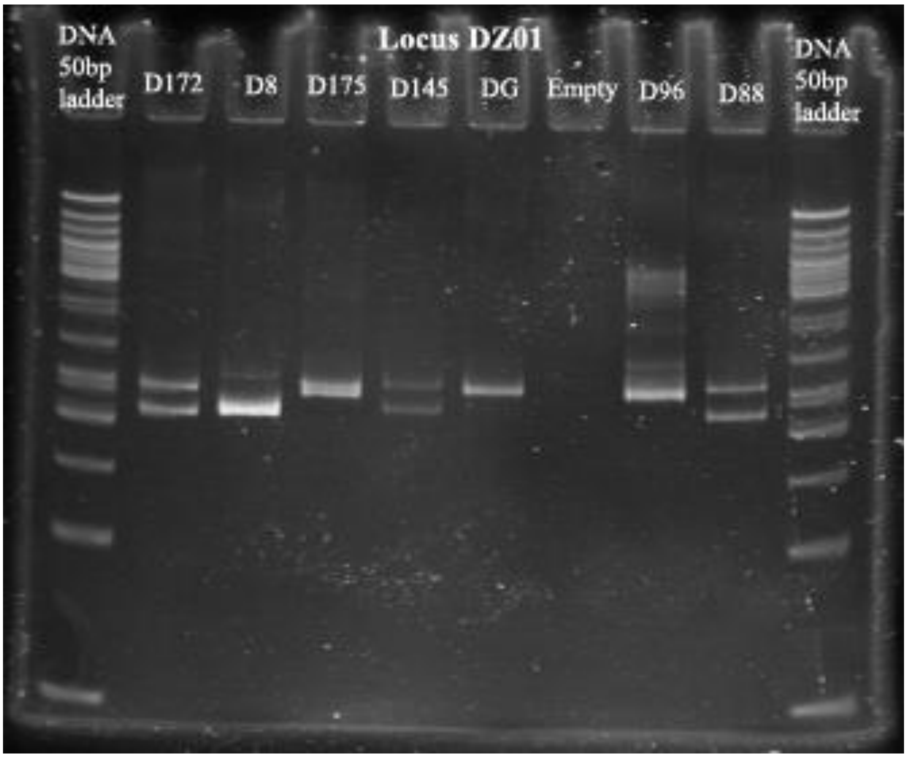

Figure S3 PCR products of seven durian types at locus DZ01 on 8% (w/v) polyacrylamide gel

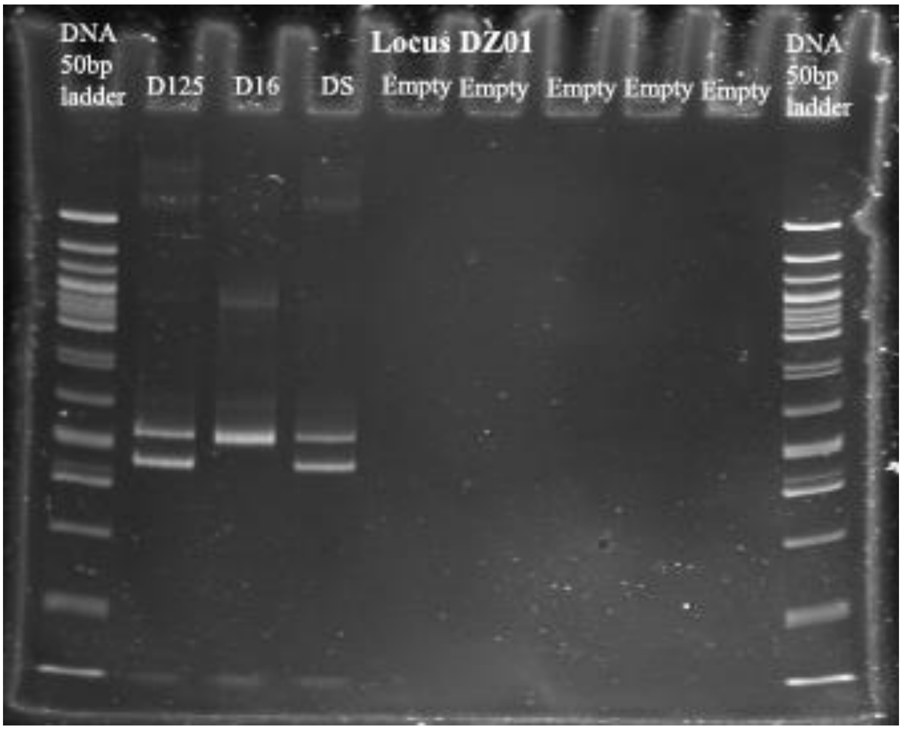

Figure S4 PCR products of three durian types at locus DZ01 on 8% (w/v) polyacrylamide gel

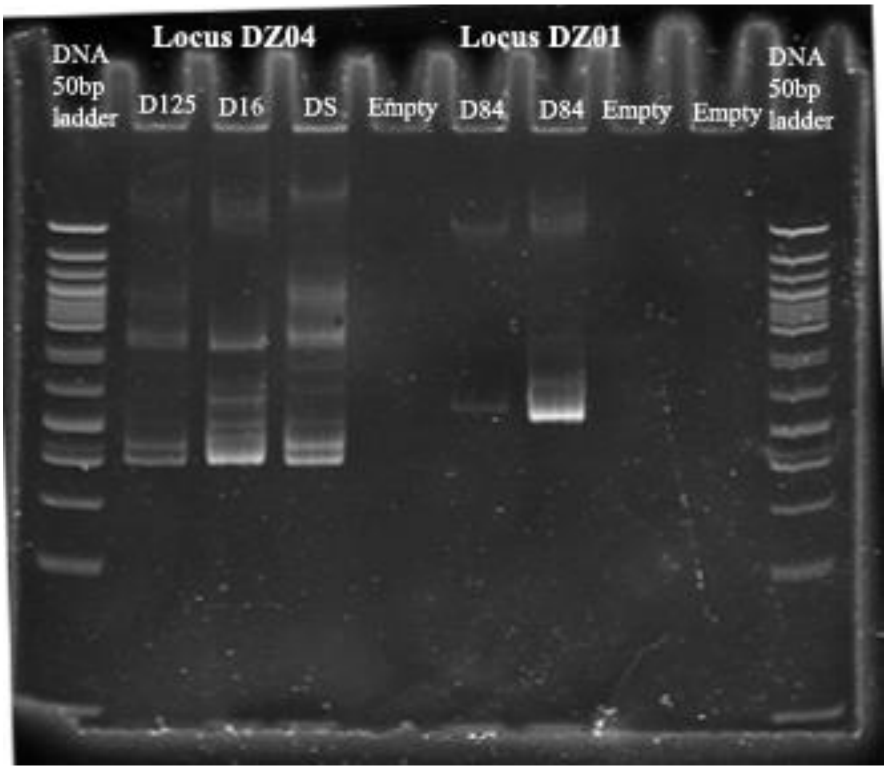

Figure S5 PCR products of two durian types and three durian types at loci DZ01 and DZ04 respectively on 8% (w/v) polyacrylamide gel

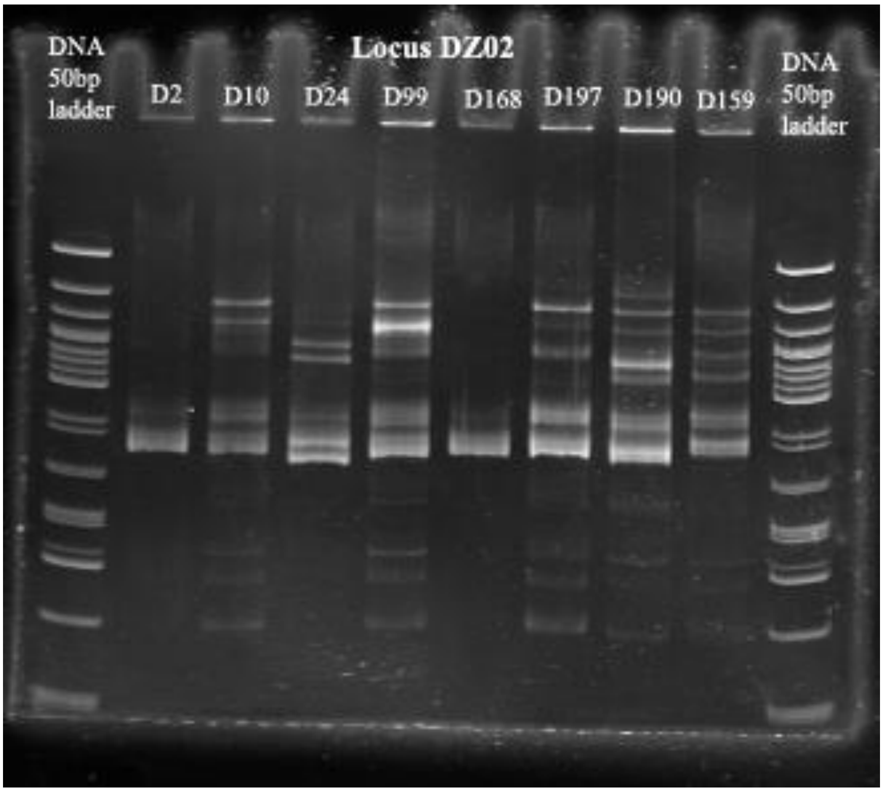

Figure S6 PCR products of eight durian types at locus DZ02 on 8% (w/v) polyacrylamide gel

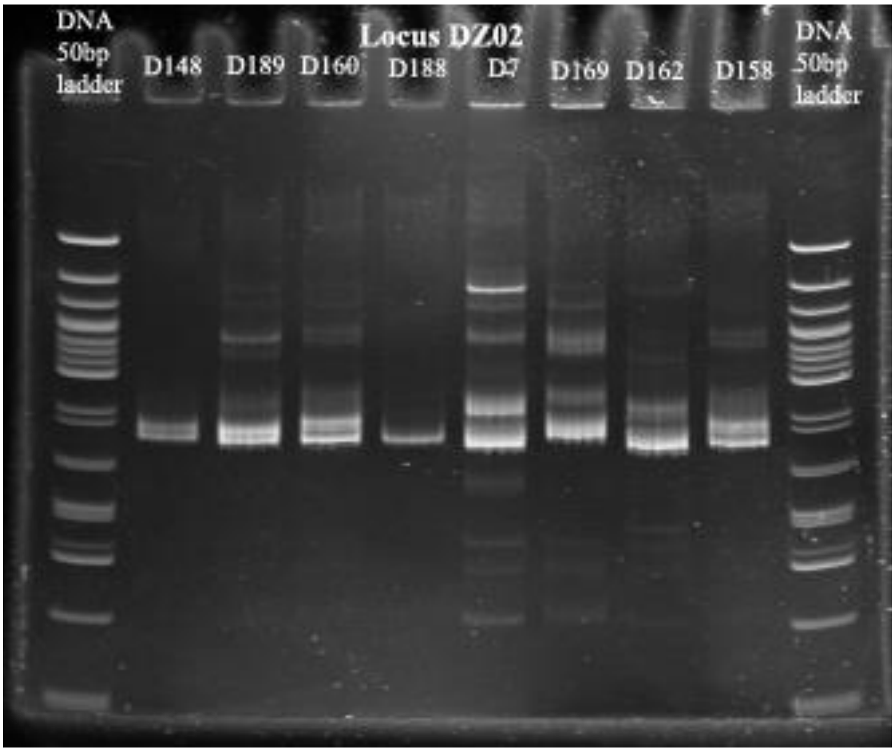

Figure S7 PCR products of eight durian types at locus DZ02 on 8% (w/v) polyacrylamide gel

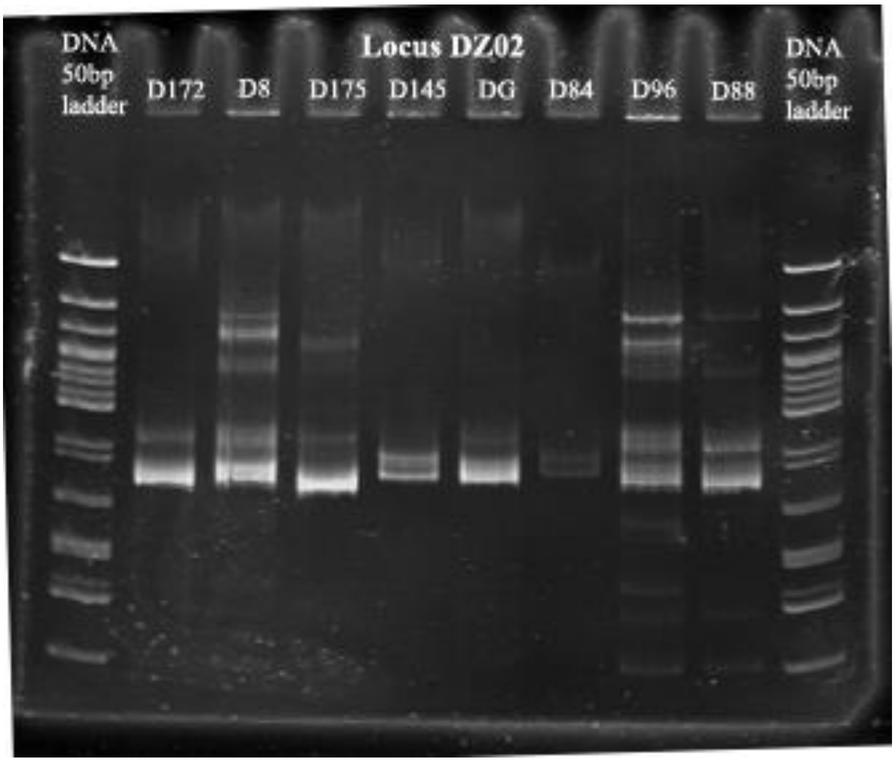

Figure S8 PCR products of eight durian types at locus DZ02 on 8% (w/v) polyacrylamide gel

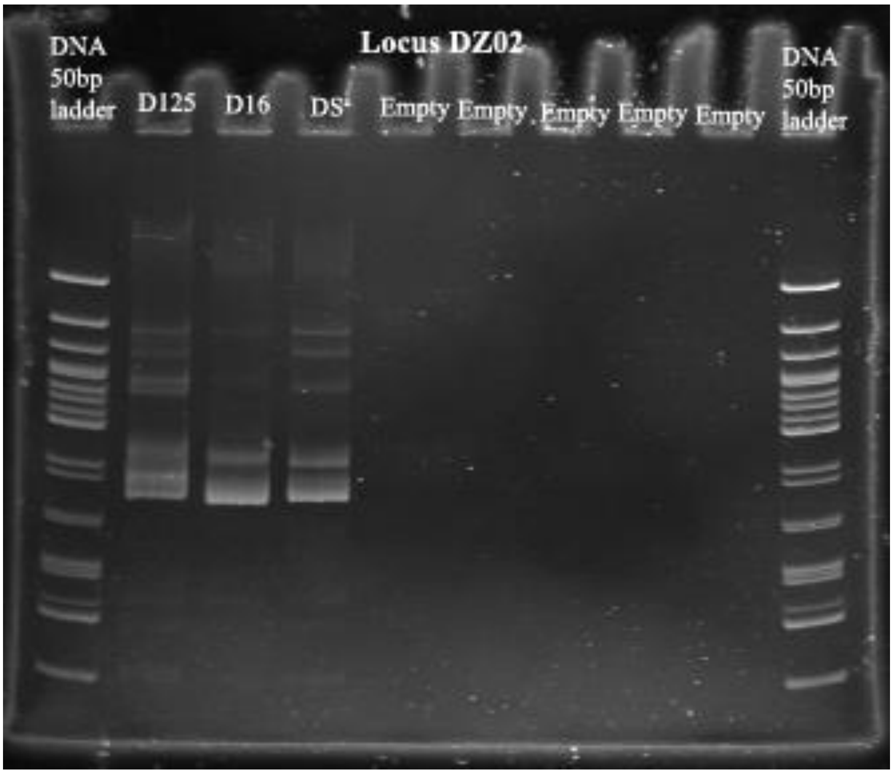

Figure S9 PCR products of three durian types at locus DZ02 on 8% (w/v) polyacrylamide gel

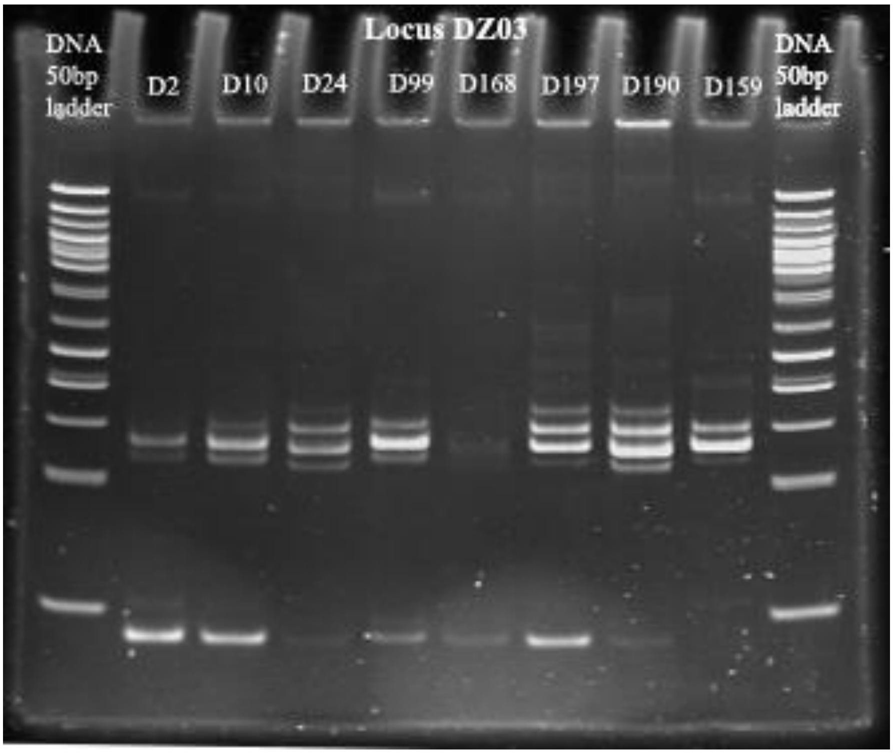

Figure S10 PCR products of eight durian types at locus DZ03 on 8% (w/v) polyacrylamide gel

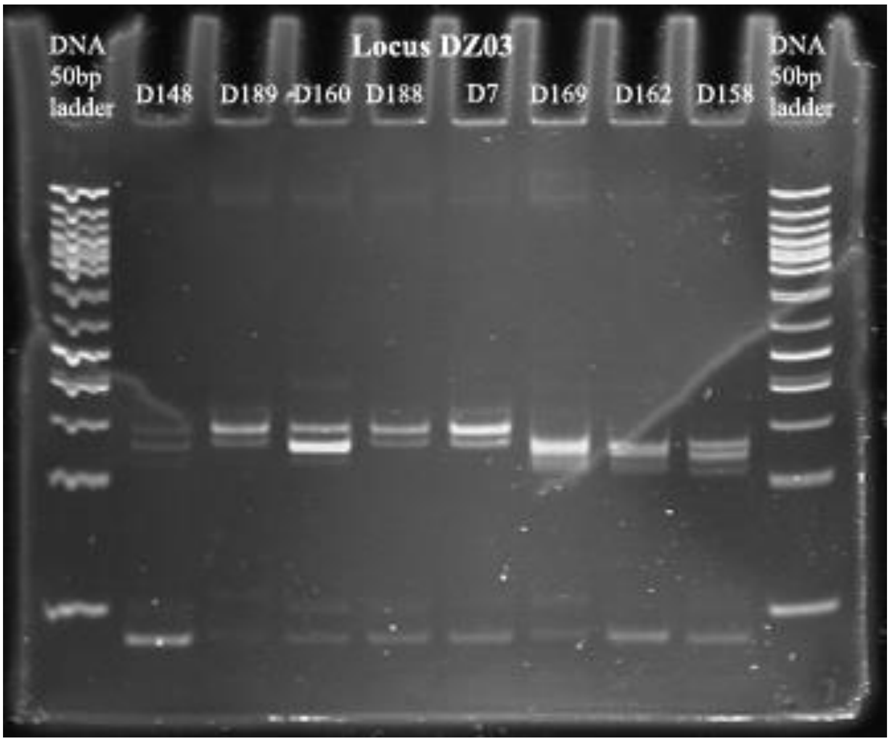

Figure S11 PCR products of eight durian types at locus DZ03 on 8% (w/v) polyacrylamide gel

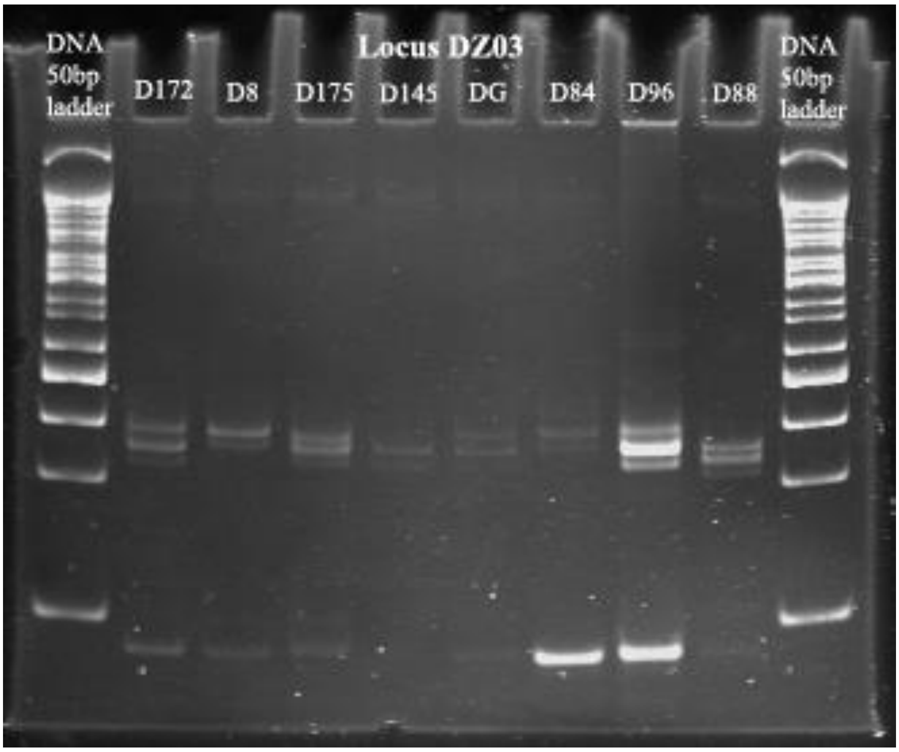

Figure S12 PCR products of eight durian types at locus DZ03 on 8% (w/v) polyacrylamide gel

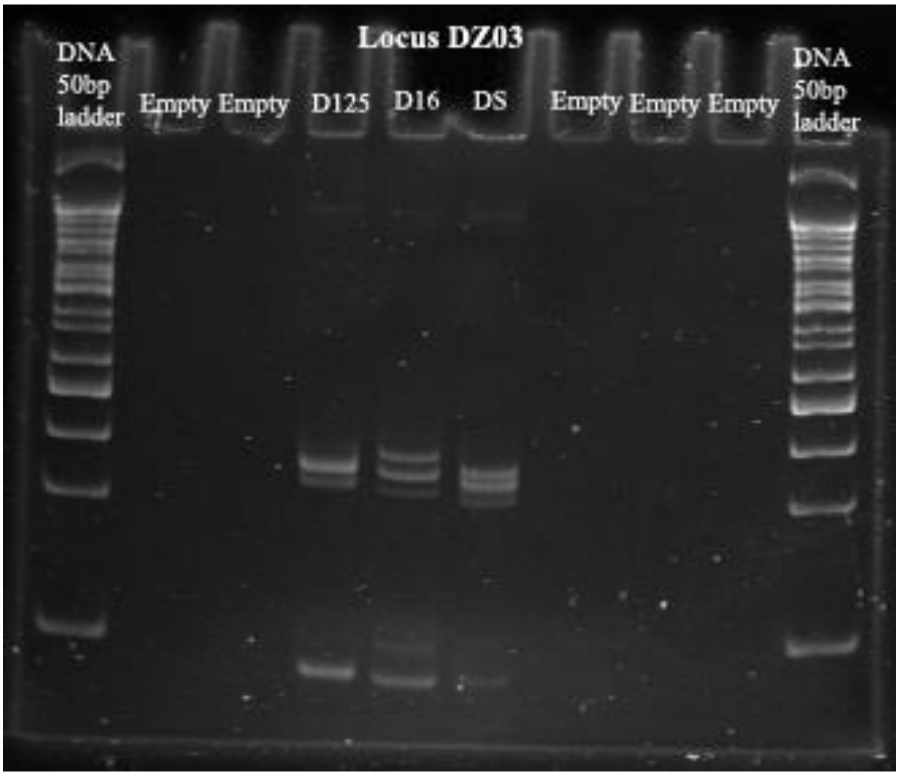

Figure S13 PCR products of three durian types at locus DZ03 on 8% (w/v) polyacrylamide gel

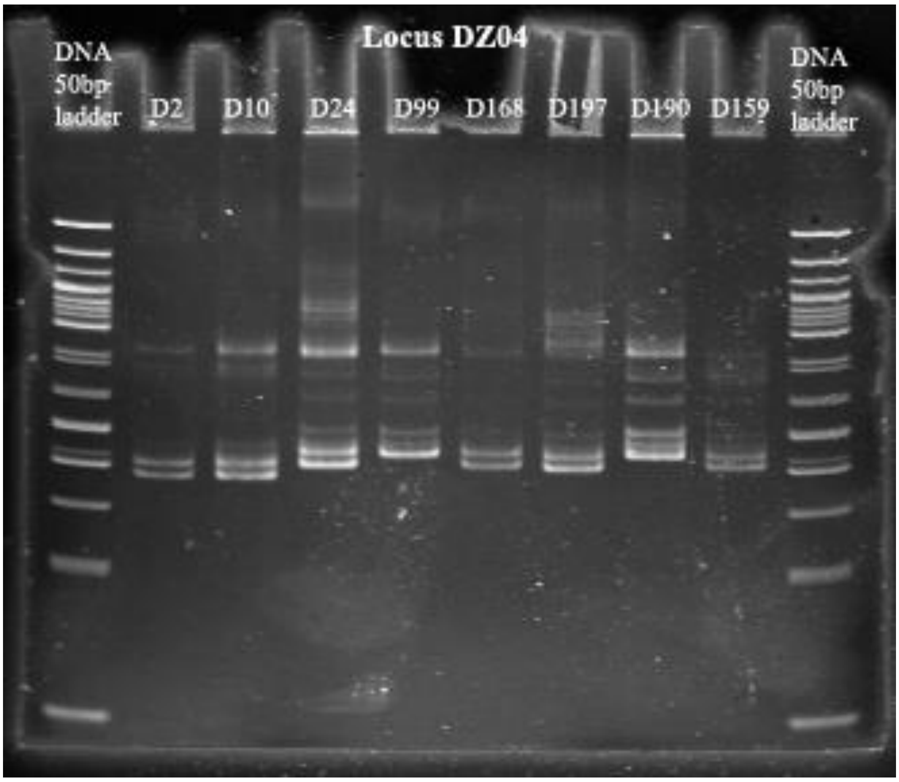

Figure S14 PCR products of eight durian types at locus DZ04 on 8% (w/v) polyacrylamide gel

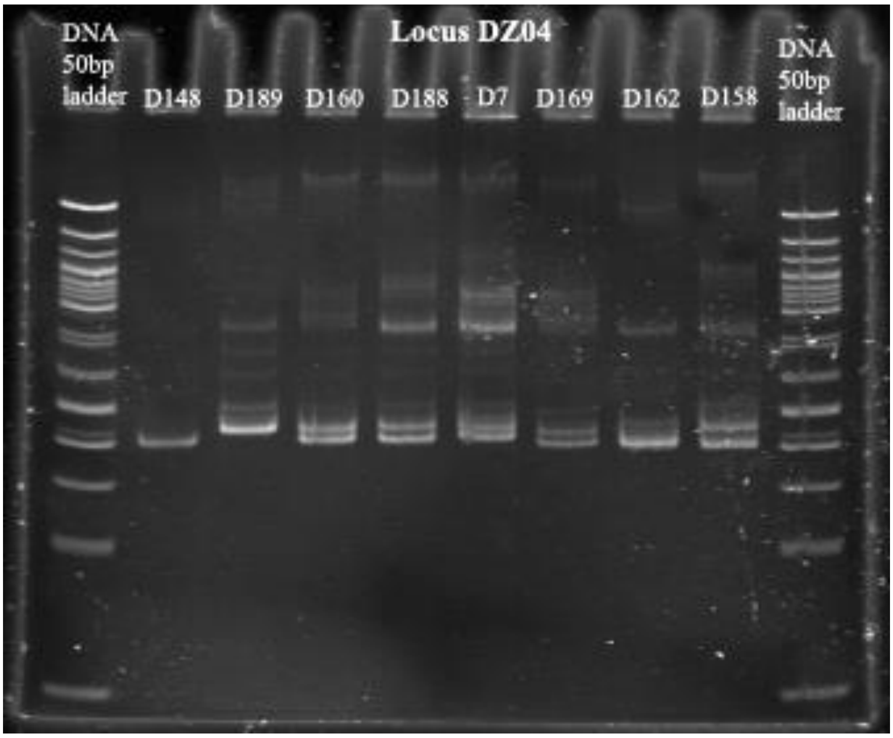

Figure S15 PCR products of eight durian types at locus DZ04 on 8% (w/v) polyacrylamide gel

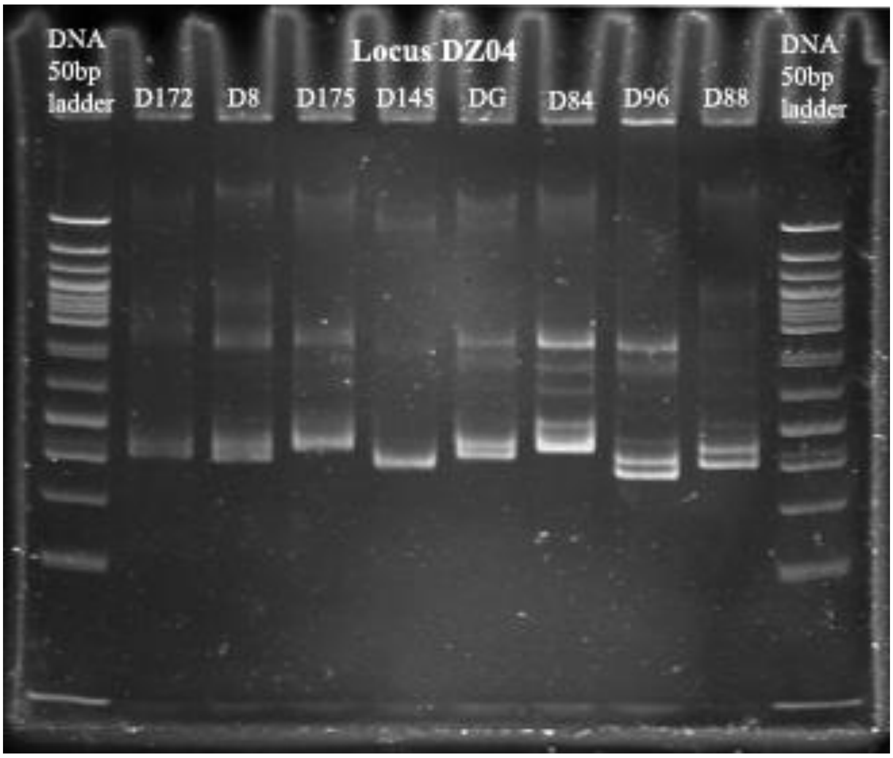

Figure S16 PCR products of eight durian types at locus DZ04 on 8% (w/v) polyacrylamide gel

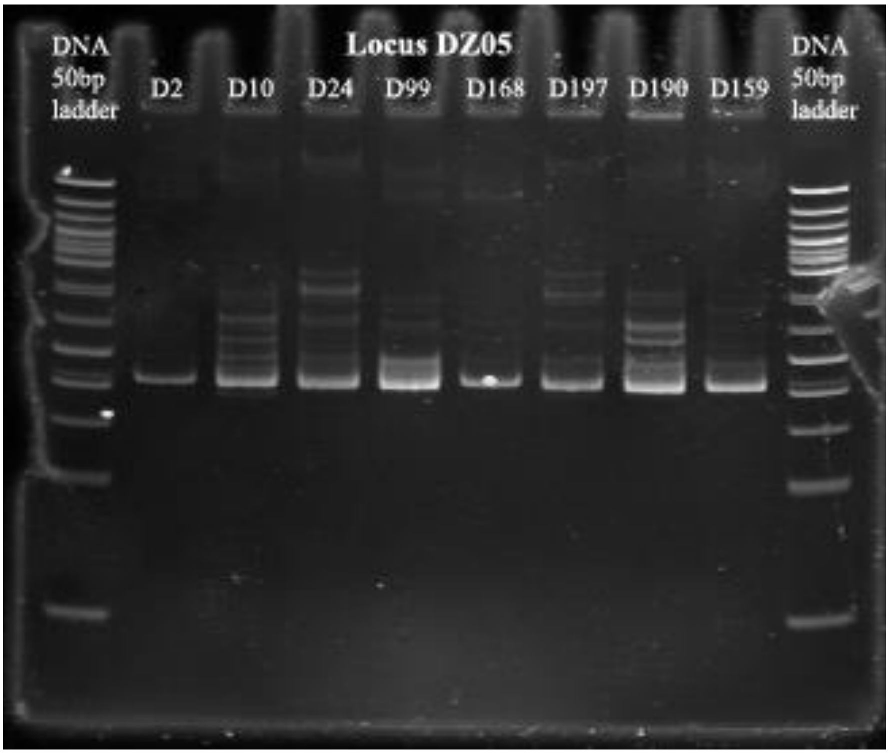

Figure S17 PCR products of eight durian types at locus DZ05 on 8% (w/v) polyacrylamide gel

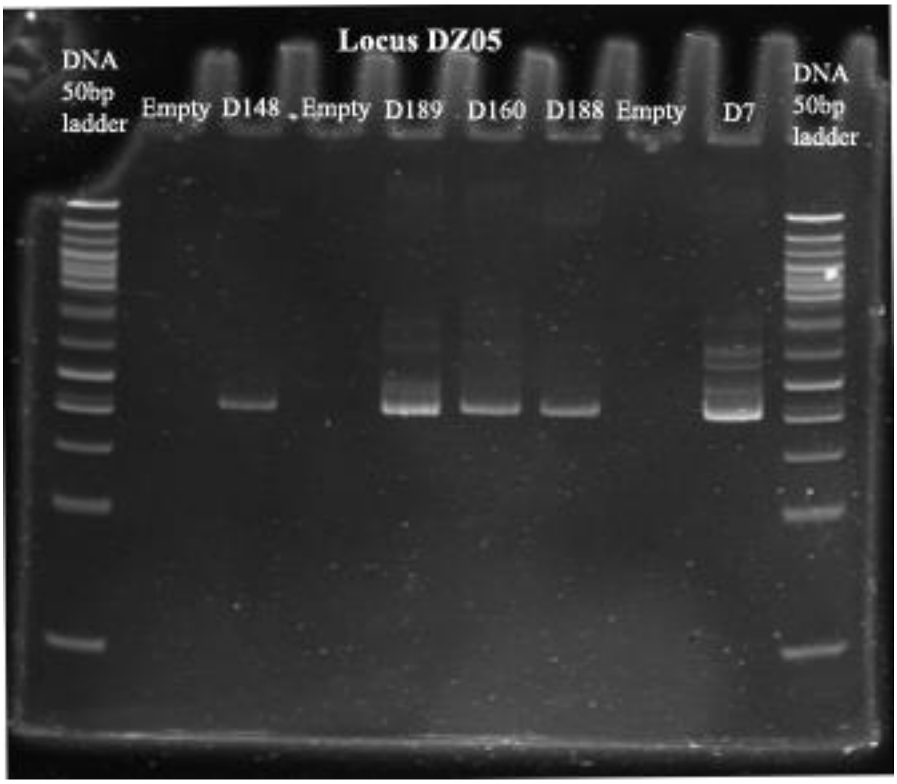

Figure S18 PCR products of five durian types at locus DZ05 on 8% (w/v) polyacrylamide gel

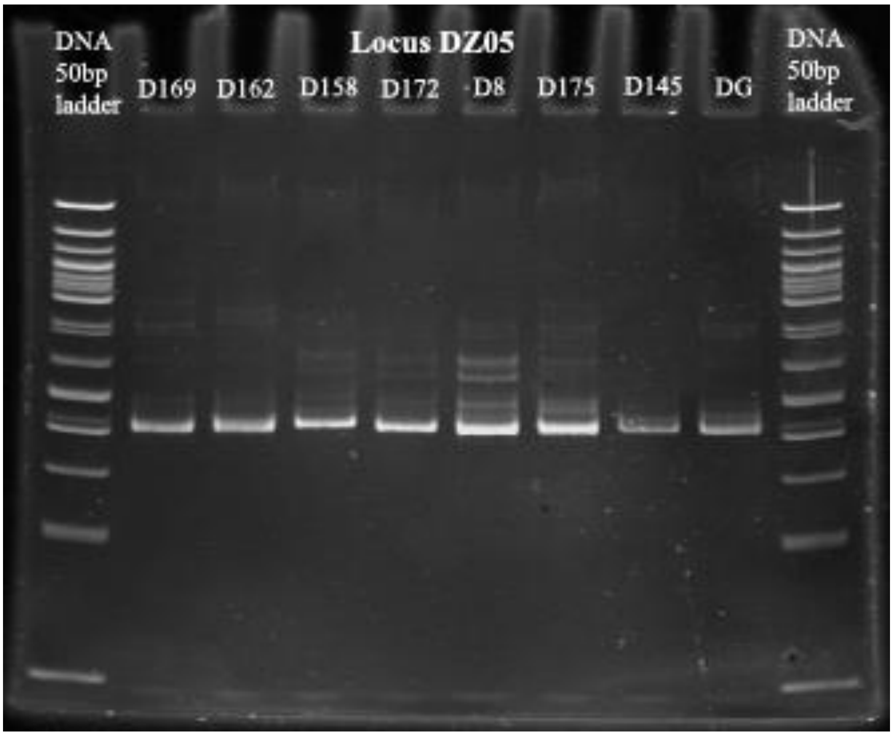

Figure S19 PCR products of eight durian types at locus DZ05 on 8% (w/v) polyacrylamide gel

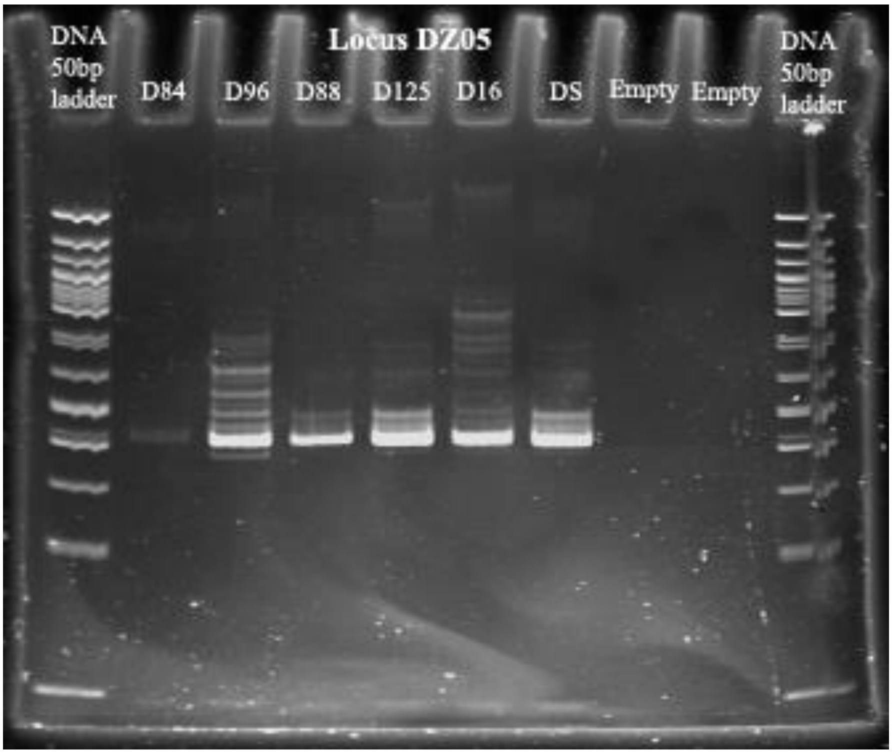

Figure S20 PCR products of six durian types at locus DZ05 on 8% (w/v) polyacrylamide gel

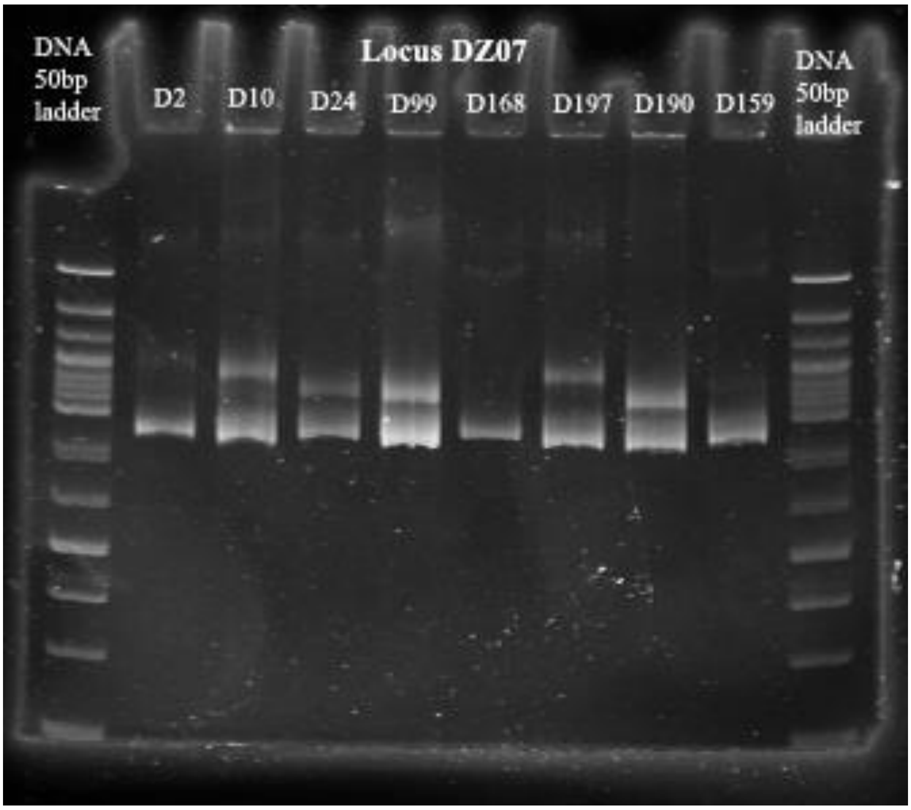

Figure S21 PCR products of eight durian types at locus DZ07 on 8% (w/v) polyacrylamide gel

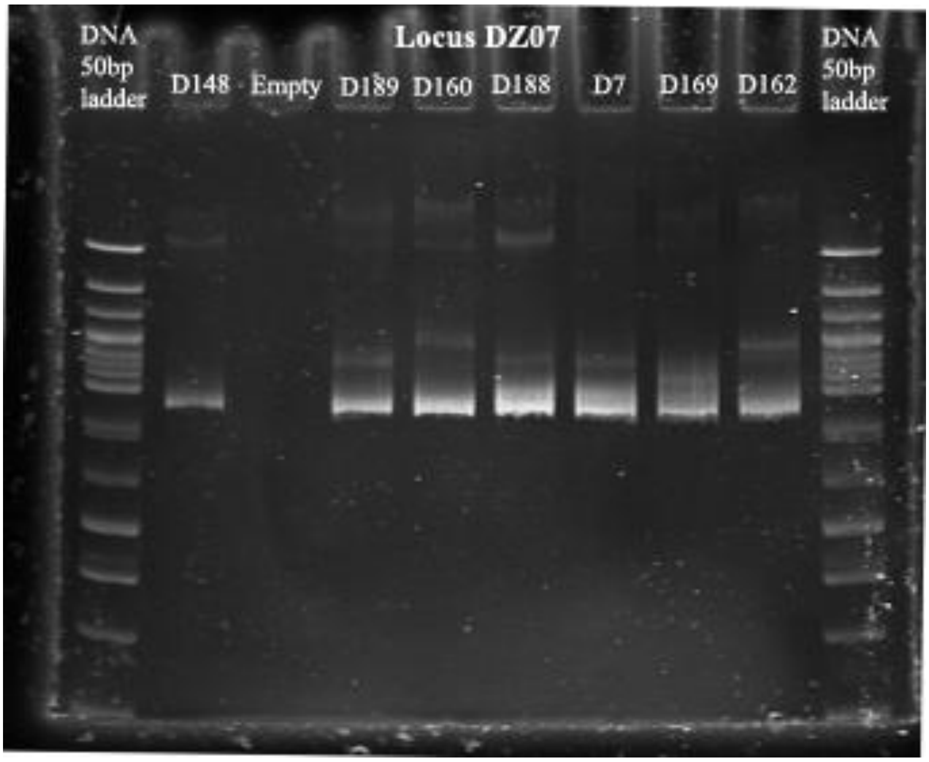

Figure S22 PCR products of seven durian types at locus DZ07 on 8% (w/v) polyacrylamide gel

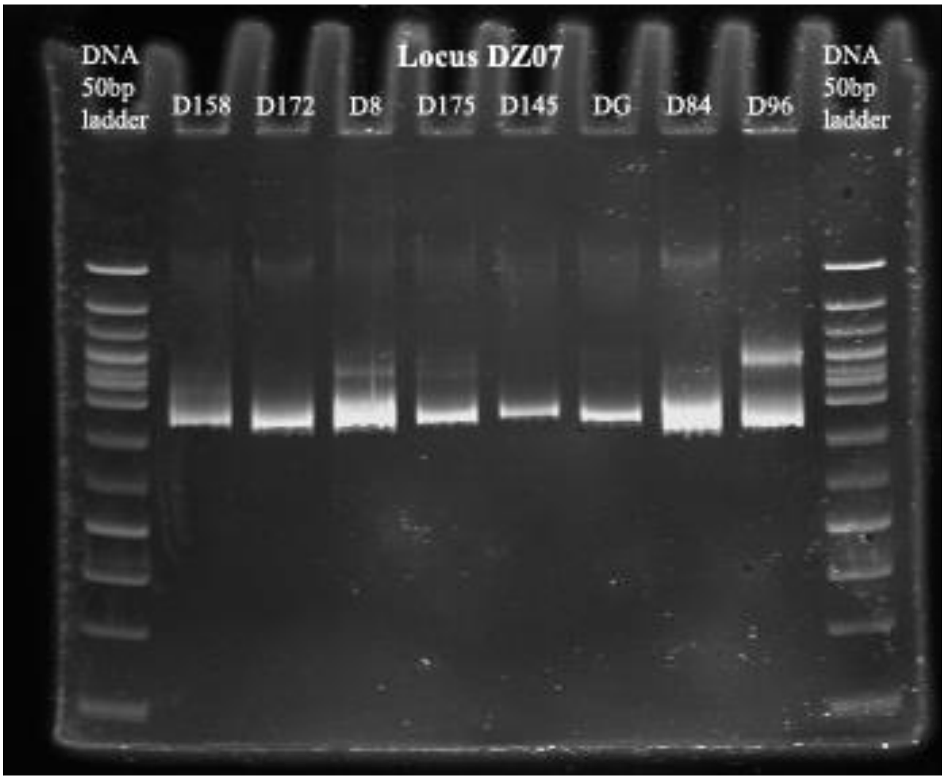

Figure S23 PCR products of eight durian types at locus DZ07 on 8% (w/v) polyacrylamide gel

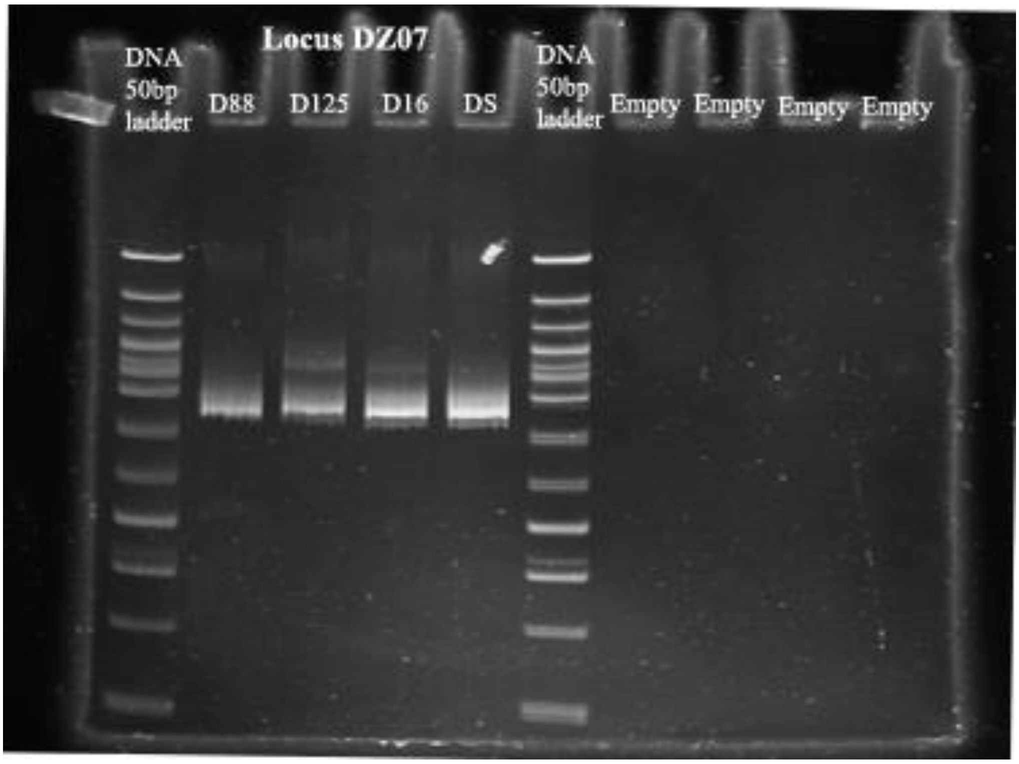

Figure S24 PCR products of four durian types at locus DZ07 on 8% (w/v) polyacrylamide gel

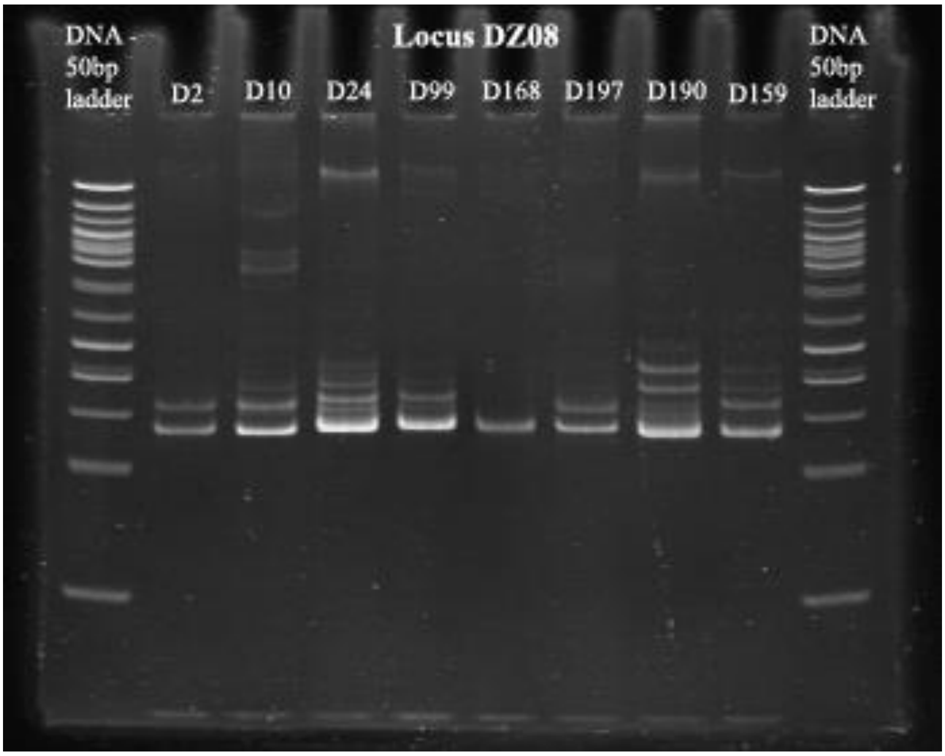

Figure S25 PCR products of eight durian types at locus DZ08 on 8% (w/v) polyacrylamide gel

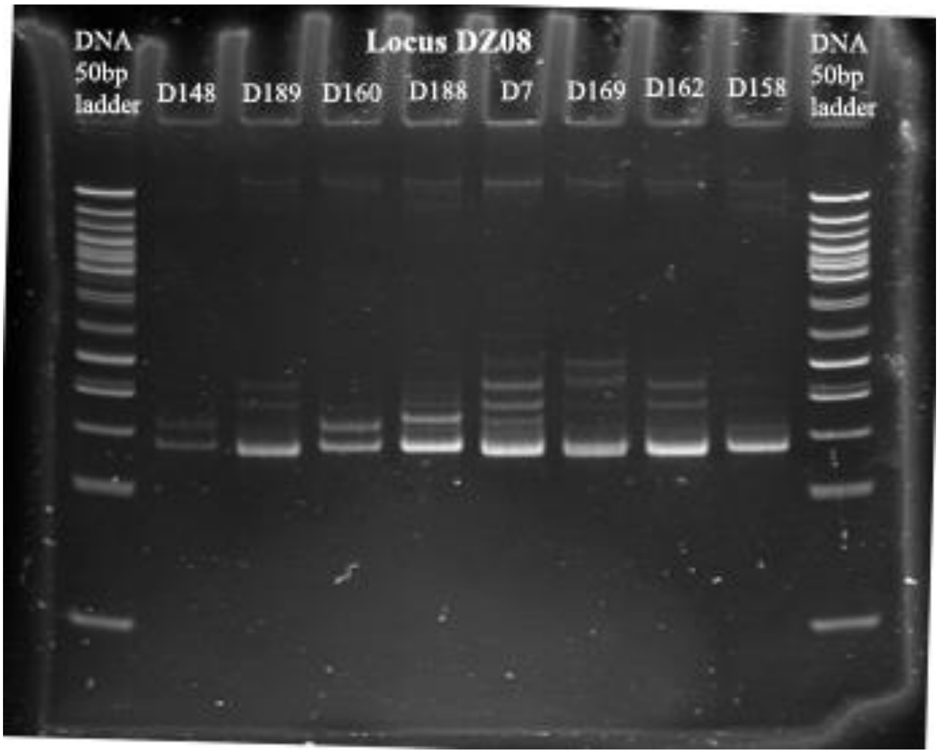

Figure S26 PCR products of eight durian types at locus DZ08 on 8% (w/v) polyacrylamide gel

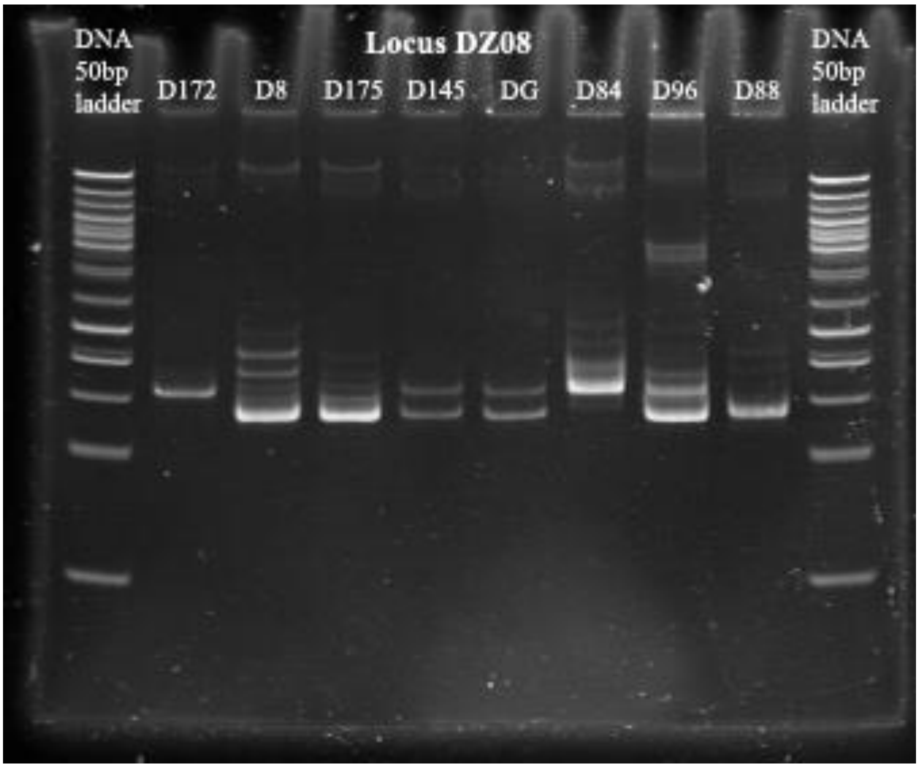

Figure S27 PCR products of eight durian types at locus DZ08 on 8% (w/v) polyacrylamide gel

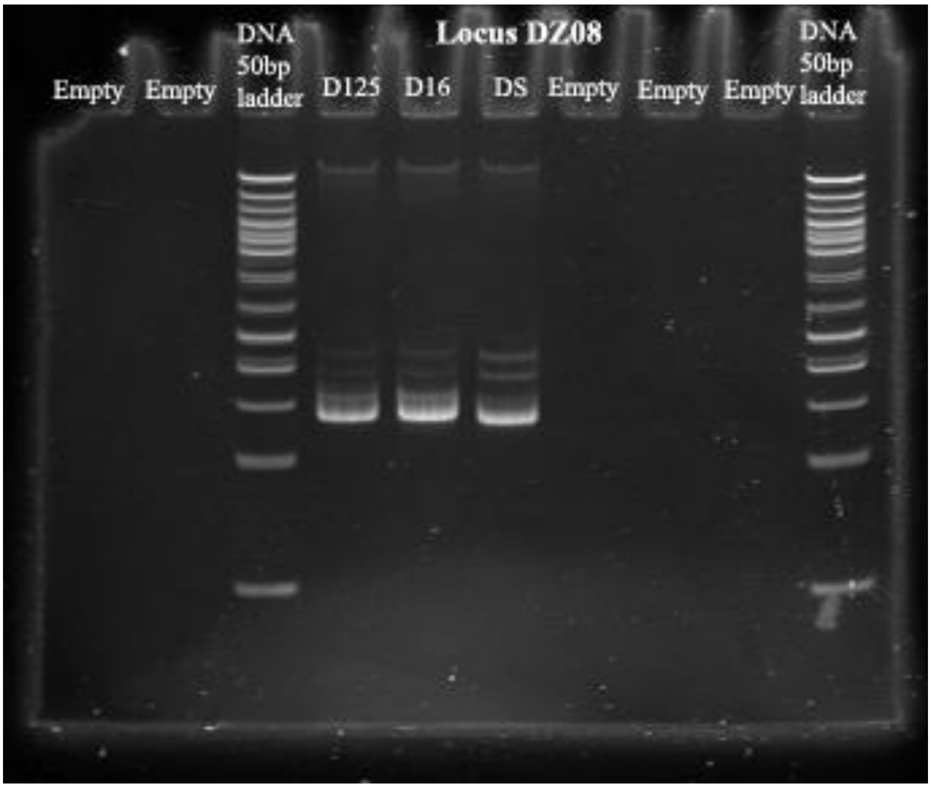

Figure S28 PCR products of three durian types at locus DZ08 on 8% (w/v) polyacrylamide gel
